# Cooperation of p300 and PCAF in the Control of MicroRNA 200c/141 Transcription and Epithelial Characteristics

**DOI:** 10.1371/journal.pone.0032449

**Published:** 2012-02-22

**Authors:** Yoshiaki Mizuguchi, Susan Specht, John G. Lunz, Kumiko Isse, Natasha Corbitt, Toshihiro Takizawa, Anthony J. Demetris

**Affiliations:** 1 Thomas E. Starzl Transplantation Institute, University of Pittsburgh Medical Center, Pittsburgh, Pennsylvania, United States of America; 2 Department of Pathology, University of Pittsburgh Medical Center, Pittsburgh, Pennsylvania, United States of America; 3 Department of Surgery, University of Pittsburgh Medical Center, Pittsburgh, Pennsylvania, United States of America; 4 Department of Molecular Anatomy and Medicine, Nippon Medical School, Tokyo, Japan; Vanderbilt University Medical Center, United States of America

## Abstract

Epithelial to mesenchymal transition (EMT) not only occurs during embryonic development and in response to injury, but is an important element in cancer progression. EMT and its reverse process, mesenchymal to epithelial transition (MET) is controlled by a network of transcriptional regulators and can be influenced by posttranscriptional and posttranslational modifications. EMT/MET involves many effectors that can activate and repress these transitions, often yielding a spectrum of cell phenotypes. Recent studies have shown that the miR-200 family and the transcriptional suppressor ZEB1 are important contributors to EMT. Our previous data showed that forced expression of SPRR2a was a powerful inducer of EMT and supports the findings by others that SPRR gene members are highly upregulated during epithelial remodeling in a variety of organs. Here, using SPRR2a cells, we characterize the role of acetyltransferases on the microRNA-200c/141 promoter and their effect on the epithelial/mesenchymal status of the cells. We show that the deacetylase inhibitor TSA as well as P300 and PCAF can cause a shift towards epithelial characteristics in HUCCT-1-SPRR2a cells. We demonstrate that both P300 and PCAF act as cofactors for ZEB1, forming a P300/PCAF/ZEB1 complex on the miR200c/141 promoter. This binding results in lysine acetylation of ZEB1 and a release of ZEB1 suppression on miR-200c/141 transcription. Furthermore, disruption of P300 and PCAF interactions dramatically down regulates miR-200c/141 promoter activity, indicating a PCAF/P300 cooperative function in regulating the transcriptional suppressor/activator role of ZEB1. These data demonstrate a novel mechanism of miRNA regulation in mediating cell phenotype.

## Introduction

The KAT3 histone acetyltransferases CREB binding protein (CBP) and P300 have at least 400 interacting protein partners, thereby acting as hubs in gene regulatory networks [1]. They are transcriptional co-activators for various sequence-specific transcription factors and play a broad biological role in cell cycle regulation, proliferation, differentiation, apoptosis, DNA damage repair, adhesion, carcinogenesis, and embryonic development [2], [3]. These molecules act primarily through acetylation of histones and other regulatory proteins (e.g. p53). Moreover, several studies suggest that disruption of P300/CBP occurs in many human diseases including cancer [4], inflammatory lung diseases [5], and viral infections [6]. These associations with human disease make P300/CBP attractive therapeutic targets.

In cancer biology, P300 is thought to be an anti-cancer gene [4]: the potential of P300 to inhibit cancer progression is linked to phenotype control, such as epithelial-mesenchymal transition (EMT). In a colon carcinoma cell line, loss of P300 induced EMT and resulted in aggressive cell migration [7]. In another study, the downstream effector of P300, *CITED2*, was shown to reduce matrix metalloproteinase-13 and inhibit cell growth in the colon carcinoma cell line RKO [8]. The underlying mechanisms, however, in these P300 anti-cancer effects need to be elucidated.

During EMT, cells acquire stem cell-like properties such as migration, invasiveness, loss of apoptosis and senescence, and immunosuppression [9]. A number of growth factors, including transforming growth factor-beta, and their downstream effectors, such as Ras and Src, are involved in EMT [9]. Loss of miR-200 family member (miR-200a,b,c, miR-141, and miR-429) expression and up-regulation of ZEB1 and ZEB2 are other important contributors to EMT [10], [11], [12].

Expression levels of miR-200 family members and ZEB1/ZEB2 are closely and inversely associated. miR-200 down-regulates the expression of the transcription factors ZEB1 and ZEB2 by binding to the 3′ untranslated region of the mRNA and preventing translation. Conversely, transcription of both miR-200 clusters, one on chromosome 1 and the other on chromosome 12, are negatively regulated by ZEB1 or ZEB2 binding to the E-box of the miR promoter [10], [11], [12]. The miR-200 family has also been described as being dysregulated during cancer progression [10], [13], and more importantly, this occurs in a stage specific manner [14].

The early stage of metastasis is similar to EMT, but later, following extravasation, metastasizing cells settle in target tissue and undergo differentiation processes that involve mesenchymal to epithelial transition (MET) [15]. According to this scenario, when cancer cells become invasive (EMT) miR-200 may be downregulated, but during re-epithelialization of distal metastases (MET) miR-200 may be upregulated. This strongly suggests that tumor cell phenotype can be malleable, possibly responsive to environmental cues. Understanding how miR-200 family expression is controlled during these processes, and how its expression affects phenotype, can yield clarification of cancer progression as well as potential therapeutic targets.

To address these questions, we generated an *in vitro* model of EMT, obtained by stable transfection of HuCCT-1 cholangiocarcinoma cells with one of the epidermal differentiation complex genes, small proline rich protein (SPRR) 2a. Over expression of SPRR2a causes HuCCT-1 cells to undergo EMT, as indicated by loss of E-cadherin, upregulation of vimentin, induction of cell motility and a change in cell morphology [16]. While investigating SPRR2a-induced EMT, we found that acetylation enhanced miR200c transcription and moderated expression levels of some EMT markers in our stable transfectants. We show that the acetyltransferases P300 and PCAF activate miR200c/141 transcription by interacting at its promoter region via the cysteine-histidine rich (CH3) domain of P300. Furthermore, these acetyltransferases overcome ZEB1 transcriptional suppression of miR200c, most likely through lysine acetylation of ZEB1. Our data shows the importance of the CH3 domain of P300 in regulating miR200c expression and the relationship to epithelial/mesenchymal status.

## Materials and Methods

### Mice, cultured cells, and SPRR2a stable transfectants

The human intrahepatic cholangiocarcinoma cell line HuCCT-1 was cultured as described [10], [17]. To evaluate the effects of SPRR2a expression, we made stable transfectants with a SPRR2a expressing vector as previously reported [16]. The negative control was a stable transfectant containing the vacant vector.

### TGF-β1, TSA/AZA, and PP2 treatment of cells

Cells were plated and cultured for 24 hours prior to treatment with 1 µg/ml of 5Aza-dC(AZA) (Sigma, St. Louis, MO) or 1 ug/ml of Trichostatin A (TSA) (Sigma) for 48 hours [18]. For the combination AZA/TSA, cells were dosed first with 1 ug/ml of AZA for 24 hr followed by treatment with 1 ug/ml of TSA for an additional 24 hr. For TGF-β1 treatment, cells were plated for 8–12 hours, washed with PBS and incubated in serum free medium (SFM) for 8 hrs. Recombinant human TGF-beta1 (5 ng/ml, R&D, Minneapolis, MN) was added to the SFM and cells incubated for an additional 24 hrs. Finally, sub-confluent 24 hour cell cultures were treated with 20 µM PP2 (Calbiochem, San Diego, CA) for designated times.

### siRNA, Pre-miR and anti-miR transfections

Cultured cells were transfected with: target specific (ABL1; Assay ID s866, EP300; Assay ID s4696,) or negative control Silencer® Select siRNA; precursor hsa-miR-200c and hsa-miR-141 (ID: PM11714; PM10860); Anti-miR™ 200c and 141 inhibitors (ID: MH11714; MH10860) (Ambion, Austin, TX) for 8 hours in serum free medium. Serum supplemented medium was added and gene and protein expression measured at the indicated time points.

### Biotinylated oligonucleotide precipitation Assays

The assays were carried out as described [19]. Briefly, 24 hours after transfection, cells were lysed with HKMG buffer (10 mM HEPES, pH 7.9, 100 mM KCl, 5 mM MgCl2, 10% glycerol, 1 mM DTT, and 0.5% of NP-40) containing protease and phosphatase inhibitors. Extracted proteins were pre-cleared with ImmunoPure streptavidin-agarose beads (Pierce, Rockford, IL) for 1 hr. Pre-cleared lysates were then incubated 12 hours with 1 µg of the 5′-biotinylated double-stranded oligonucleotides and 10 µg of competitor DNA (poly (dI-dC).poly(dI-dC) to eliminate non-specific protein/DNA interactions. Oligo-specific bound proteins were collected with streptavidin-agarose beads and separated by SDS-PAGE. Protein identification was done by Western blotting.

### Transfection and luciferase reporter assay

Transfections with DNA plasmids and siRNA molecules were done with Lipofectamine 2000 (Invitrogen, Carlsbad, CA) and with Lipofectamine RNAiMAX (Invitrogen), respectively, according to the manufacturer's instructions. Luciferase assays were carried out with a Promega (Madison, WI) assay kit system and measured on a luminometer.

### Western blotting and Immunoprecipitation

For Western blotting, cell lysates were obtained using TNE buffer (50 mM Tris pH 8.0, 150 mM NaCl, 10% v/v NP40, 2 mM EDTA) containing protease inhibitors. All protein concentrations were measured using a BCA Protein Assay kit (Pierce). Proteins were separated by SDS-PAGE, transferred to nitrocellulose membranes, and blocked with 5% skim milk in TBST (Tris-buffered Saline Tween-20). Standard immunostaining was carried out using enhanced chemiluminescence reagents (Pierce).

For immunoprecipitation, TNE cell lysates were incubated with appropriate antibodies and the protein/antibody complexes collected using protein G Dynabeads (Invitrogen). Immunoprecipitated proteins were identified by Western blotting. The antibodies used in this study are as follows: anti-HA antibody (F-7, Santa Cruz, Santa Cruz, CA); anti-P300 antibody (N-15, Sata Cruz); anti-ZEB1 antibody (H102, Santa Cruz); anti-PCAF (C14G9, Cell Signaling Technology, Danvers, MA); anti-acetylated lysine (9441, Cell Signaling Technology); anti-Flag (M2, Sigma); anti-S100A4 (X9-7, Santa Cruz); anti-vimentin (BioVision, Mountain View, CA); anti-E-cadherin (36, BD Biosciences, Bedford, MA); anti-V5 (Invitrogen)

### Immunostaining

Cells were cultured on glass coverslips and transfected with appropriate agents as described above. At the designated time point cells were fixed for 1 hour in 1% paraformaldehyde prior to staining. Primary antibody: E-cadherin (listed above); secondary antibody: biotinylated horse anti-mouse (Vector Laboratories, Burlingame, CA). Fluorescence was obtained using streptavidin conjugated Qdots® (Invitrogen).

### RNA and genomic DNA extraction, PCR, and real-time PCR

Total RNA was extracted using Trizol (Invitrogen) according to the manufacturer's instructions. Reverse transcription was performed using a High Capacity cDNA reverse transcription kit (Applied Biosystems, Foster City, CA) and random primers. Total genomic DNA was extracted using PureLink Genomic DNA Mini Kit (Invitrogen). To amplify the target genes, PCR reactions were performed with EX Taq Hot Start polymerase (Takara Bio Inc, Otsu, Shiga, Japan). PCR products were analyzed on 12% polyacrylamide gels and visualized using ethidium bromide. Gene expression of mRNA, primary-miRNA, and precursor- miRNA were quantified by SYBR Green or TaqMan real-time PCR using specific primers. The real-time SYBR Green PCR primers were designed using Primer 3 software and are as follows: pri-141/200C, for- AGGGAAGGGGTTAAGGCAGT and rev- GAGGTGCCTAGGGAACCAGT; E-cadherin, for- CCCACCACGTACAAGGGTC and rev- CTGGGGTATTGGGGGCATC; MLH, for- ACAGCTGATGGAAAGTGTGCAT and rev- ATTGCCAGCACATGGTTTAGG; GAPDH, for- ACAGTCAGCCGCATCTTCTT and rev- ACGACCAAATCCGTTGACTC; S100A4, for- GATGAGCAACTTGGACAGCA and rev- CTTCCTGGGCTGCTTATCTG. Expression was normalized to GAPDH using the comparative 2-ΔΔCT method. TaqMan primers were used to quantify miRNA, vimentin, ZEB1 and ABL1 expression. miRNA expression was normalized to U6 snRNA and mRNA to GAPDH using the comparative 2-ΔΔCT method (Applied Biosystems: miR-200c, 002300; miR-141, 000436; miR-200b, 002251; miR-429, 001024; U6, 001973; GAPDH, 4310884E; ABL1, Hs01104728; VIM, Hs00185584; ZEB1, Hs00232783)

### Plasmids

The human SPRR2a expression vector (pTracer) (Invitrogen) which has a C-terminal His-V5-tag was constructed as described before [16]. Human HA-tagged P300 and its CH3 deletion protein expression vectors and the Flag-tagged PCAF vector were purchased from Addgene (Cambridge, MA). The human Halo-tagged ZEB1 vector was obtained from Promega (Madison, WI). The −126/152 miR-200c/141 promoter construct was generated by PCR according to previous reports [12], and subcloned into a pGL3 vector (Promega).

### Statistics

All statistical analyses were performed using SigmaStat software. A *P* value of <0.05 was considered statistically significant, and all tests were two-tailed. All interval values are expressed as mean ± SD. Comparison between two groups was performed using the unpaired Student *t*-test. And comparison among three or more groups was performed using a one-way ANOVA.

## Results and Discussion

### Deacetylase inhibitor TSA can enhance expression of some Epithelial Markers in SPRR2a Cells

**Figure 1:**
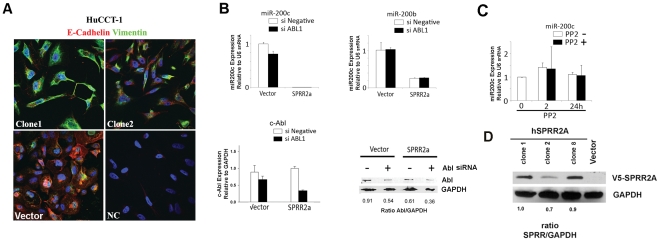
EMT induced by SPRR2a in HuCCT-1 involves loss of E-cadherin, increased vimentin, and reduction of miR-200 family transcription as compared to vector transfected controls. Examples of the morphological changes and changes in E-cadherin and vimentin expression in stable SPRR2a clones (NC = negative control) (**A**). Transcriptional loss of the miR200 family in SPRR2a expressing cells does not involve SH3 domain containing tyrosine kinases. Real-time PCR analysis of miR-200 family after 72 hrs treatment with ABL1 siRNA (**B**) and PP2 treatment (**C**) did not alter miR-200 expression. All clones used in this paper stably express SPRR2a (**D**). Real time PCR analysis: comparative 2-ΔΔCT method (miRNA: U6 internal control; ABL1: GAPDH internal control). (n = 2 independent experiments).

SPRR2a induction of EMT fully converts cells from an epithelial to mesenchymal status, as evidenced by gross morphological changes, loss of E-cadherin and upregulation of vimentin (**Figure 1A**). In addition, expression of the miRNA-200 family is strongly inhibited in HuCCT-1 SPRR2a cells, especially miR-200c (**Figure 1B**). Both clusters of the miRNA 200 family are polycistronic transcripts coded on chromosome 12 (miR-200c/141) and chromosome 1 (miR-200b/a/429) [10], [11]. The promoter region of miR-200b/a/429 has a CpG island, whereas miR-200c/141 does not [11]. Because SPRR2a can act as a SH3 ligand for Src-family kinases and Abl-family kinases, and SPRR2a expression results in Src activation (418Tyr phosphorylation) [16], we hypothesized that SH3 domain containing tyrosine kinases might play a role in SPRR2a induced EMT. Therefore, we first examined the effect of siRNA molecules targeting ABL1 on miRNA-200c expression in HuCCT-1-SPRR2a cells (**Fig. 1B**). Since this did not affect miR200c or miR200b expression, we next examined the effect of a more global tyrosine kinase inhibitor, PP2, with the expectation that PP2 would increase miR-200c and shift cells towards an epithelial phenotype. Real-time PCR analysis showed that PP2 does not affect miRNA-200c levels (**Figure 1C**).

We next tested whether the acetylation and/or methylation status were important in controlling miR-200 family expression by treating SPRR2a cells with the histone deacetylase inhibitor, Trichostatin A (TSA), and the methyltransferase inhibitor, 5-aza-2′-deoxycytidine (AZA). Individually, these agents are used to determine the relevance of acetylation or de-methylation in gene expression. If used together, TSA and AZA often yield transcriptional synergistic effects, a tool frequently employed to unmask epigenetically silenced genes in malignancies. Acetylated histones lose their charge, enabling DNA unwinding, and unmethylated DNA allows transcription factors easier access to promoter regions. In combination, they remove major conformational impediments to gene transcription.

**Figure 2:**
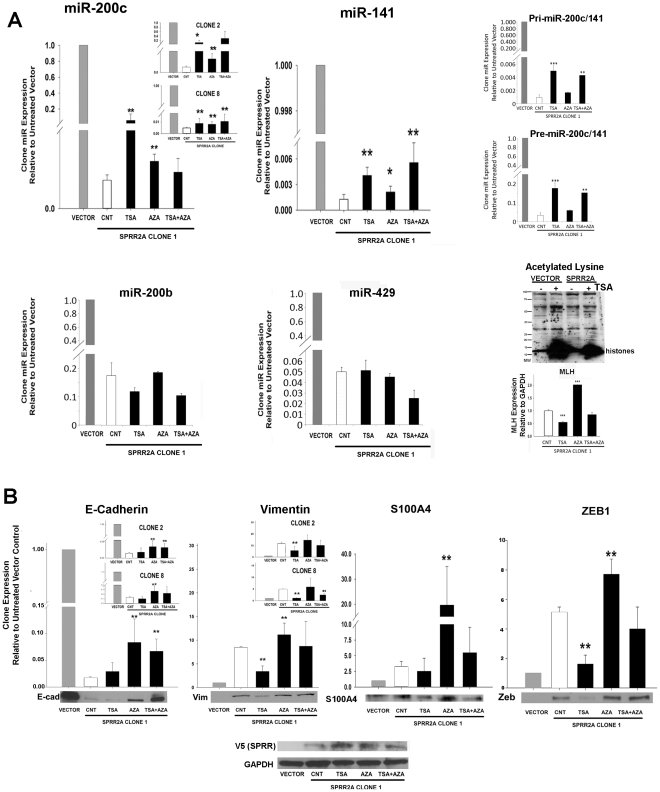
In mesenchymal HuCCT-1 SPRR2a cells, acetylation can cause epithelial shifts in miR200c/141, vimentin and Zeb1 expression. De-methylation has less impact on miR200c/141 transcription, but does affect EMT marker protein expression. In HuCCT-1 SPRR2a cells, TSA yields a greater epithelial shift in miR200c/141 expression than AZA, while miR200b/a/429 is unaffected (**A**). TSA increased expression levels for both primary miR-200c/141 and premature miR-200c transcripts as well (**A**). The efficacy of TSA/AZA treatment was monitored by western blot for acetylated lysine (TSA) and real time PCR for MLH expression (AZA) (**A**). Expression levels of EMT markers (both mRNA and protein) showed that TSA also reduced expression of vimentin and Zeb1 in SPRR2a cells (partial MET shift), while AZA treatment increased expression of all markers tested (**B**). Finally, SPRR2a over expression was maintained during TSA/AZA treatment (**B**). Real time PCR analysis: comparative 2−ΔΔCT method (U6 or GAPDH internal control) and expression levels for HuCCT-1-vector cells were set to 1.0. (n≥3 independent experiments; *, *P*<0.05; **, *P*<0.01; ***,*P*<0.005; Student's *t*-test).

Treatment of SPRR2a expressing cells with TSA significantly increases expression levels of miR-200c and miR-141, but had little effect on miR429/200b (**Figure 2A**). To confirm that TSA is activating miR-200c transcription, we show a similar increase in expression levels for both primary miR-200c/141 and premature miR-200c transcripts (**Figure 2A**). SPRR2a cells show some increase in miR200c/141 expression after AZA treatment as well, indicating that this gene is also regulated in part by DNA methylation [20]. However, the impact on miR200c/141 transcription was greater following TSA treatment. The efficacy of our treatments was monitored by examining changes in lysine acetylation after TSA via western blot and by measuring MLH gene expression after AZA treatment, a gene whose expression is known to be controlled through methylation [18] (**Figure 2A**).

Since TSA and AZA partially recovered epithelial miR200c expression, we then examined their influence on expression of downstream EMT-associated genes. SPRR2a induced EMT in HuCCT-1 cells correlates with changes in E-cadherin, ZEB1, vimentin, and S100A4 expression, while other EMT-associated genes such as TWIST or SNAIL remain unchanged (gene array and real time PCR; data not shown). Concordant with TSA reversal of miR-200c expression, there was significant down regulation of ZEB1 and vimentin (MET changes). E-Cadherin and S100A4 expression levels did not significantly change (**Figure 2B**), but the observed trends were in the expected direction.

EMT/MET is a complex process. SPRR2a induction causes changes in morphology and staining intensities (**Figure 1**) that represent both phenotypic extremes: an epithelial (vector) or mesenchymal (SPRR2a clone) state, both of which correlates with the expression levels of important molecules as measured by PCR and western blots (**Figure 2**). Our interpretation of changes in the mRNA and protein levels following cell treatments represent the extent of epithelial/mesenchymal shift. Transitioning from an epithelial to mesenchymal (and visa-versa) phenotype is not an all-or-nothing event, but instead, occurs along a continuum in response to the level of signals, factors and co-factors within the cell [21]. Consequently, TSA contributes to MET changes in SPRR2a cells (increased miR200c; decreased Vimentin and ZEB1), but not to the same extent seen in the vector cells and alone was unable to completely reverse the gross (visible) cell phenotype (data not shown). This data does, however, emphasize the role of acetylation in regulation of miR200c, ZEB1 and VIM. Lastly, AZA treatment increased expression of all genes tested in **Figure 2B**, indicating that expression levels for these genes (E-CAD, VIM, S100A4, ZEB1) can be regulated by CpG methylation.

To verify that the response to TSA and AZA in our SPRR2a cells was not due to a clonal effect, we measured miR200c, E-cadherin and vimentin in two other SPRR2a stable transfectants and observed similar responses. In addition, SPRR2a expression was maintained in the clones during treatments, indicating that the results were not attributable to loss of SPRR2a expression. If anything, TSA slightly increased SPRR2a expression.

### P300/PCAF activate miR-200c/141 promoter

**Figure 3:**
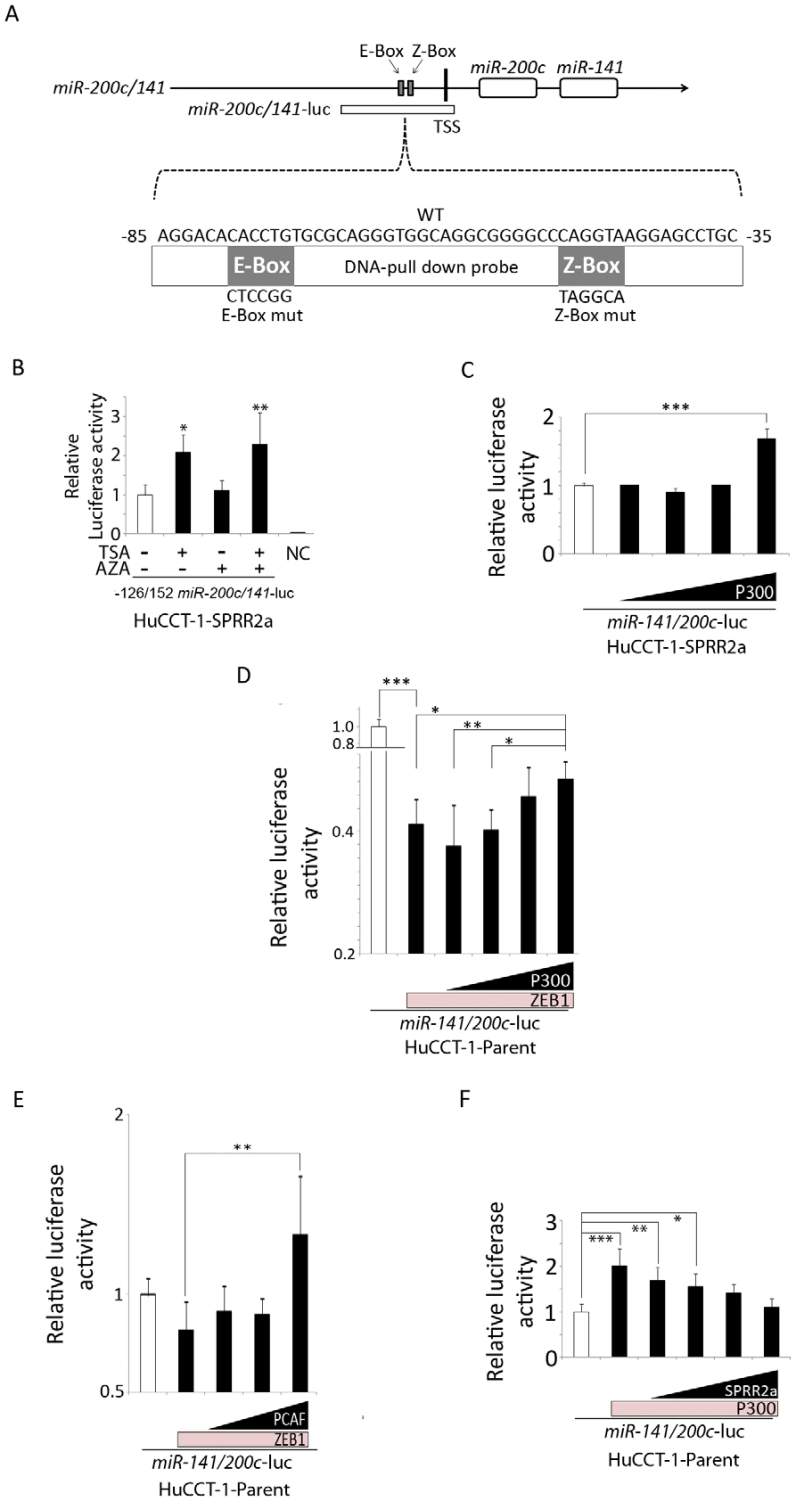
P300 and PCAF activate the *miR-200c/141* promoter, while ZEB1 and SPRR2a inhibit this activation. Illustration of *miR-200c/141* promoter, E-box, Z-box, and transcription starting site (TSS) as well as *miR-200c/141*-luciferase vector and DNA-pull down assay probes (**A**). Luciferase assay for *miR-200c/141*-promoter activity in SPRR2a expressing cells: Treatment with TSA and/or AZA shows TSA increased promoter activity (**B**), as did transfection with a P300 expression vector (0, 0.05, 0.1, 0.2, 0.4 µg) (**C**). Luciferase assay for *miR-200c/141*-promoter activity in HuCCT-1 parent cells: transfection with a ZEB1 expression vector (0.1 µg) reduced *miR-200c/141*-promoter activity, while co-transfection with P300 (0,0.05, 0.1, 0.2, 0.4 µg) (**D**) or PCAF (0, 0.1, 0.2, 0.4 µg) (**E**) antagonized this repression. In contrast, transfection with a P300 expression vector (0.4 µg) enhanced *miR-200c/141*-promoter activity, while co-transfection with SPRR2a (0,0.05, 0.1, 0.2, 0.4 µg) negated this effect (**F**). (Data represents 2–3 independent experiments; *, *P*<0.05; **, *P*<0.01; ***, *P*<0.001; (B) Student's *t*-test; (C–F) one-way ANOVA).

CBP/P300 participate in transcriptional control by: 1) bridging gene-specific transcription factors with the basic transcriptional component, 2) contributing to the formation of multi-protein complexes and modulating the activation status of gene-specific transcription factors through post-translational modifications and 3) exhibiting acetyl-transferase activity on nucleosomes and certain gene-specific transcription factors. Using a *miR-200c/141* promoter construct, which includes the E-box and Z-box elements (**Figure 3A**), we found that TSA treatment enhanced promoter activity (**Figure 3B**). This observation suggests that some proteins that bind to the promoter region are acetylated and this acetylation increases *miR-200c/141* promoter activity.

One such candidate protein is ZEB1. ZEB1 is involved in transcriptional control of a number of key regulatory genes involved in differentiation and development [22]. ZEB1 can act as a transcriptional repressor through recruitment of the co-repressor, C-terminal binding protein (CtBP) [23]. However, transcriptional activation of the vitamin D3 receptor and the estrogen-responsive *ovalbumin* gene by ZEB1 occurred after recruitment of P300 and PCAF to the ZEB1 binding site [24]. Binding of PCAF to ZEB1 acetylates several lysine residues close to the CtBP interacting domain of ZEB1, displacing CtBP and switching ZEB1 from a transcriptional repressor to an activator [24]. On the miR-200c/141 promoter, ZEB1 binding to the E-box and Z-box elements suppresses transcriptional activity [12]. Since ZEB1 binds the miR-200c/141 promoter and TSA-induced acetylation enhanced promoter activity, we investigated what role the acetyltransferases P300 and PCAF might have on transcription of the miR-200c/141 promoter. HuCCT-1-SPRR2a cells (low miR-200c/141; high ZEB1) transfected with a P300 vector show increasing promoter activity in a dose-dependent manner (**Figure 3C**). In parent HuCCT-1 cells (high miR-200c/141; low ZEB1), transfection of a ZEB1 expressing vector significantly inhibited the promoter activity, but high dose co-transfection with a P300 vector significantly antagonized the ZEB1-induced suppression (**Figure 3D**). A similar effect was also observed in HuCCT-1 parent cells transfected with ZEB1 and PCAF expression vectors (**Figure 3E**), indicating that both P300 and PCAF can activate the *miR-200c/141* promoter, probably through acetylation of ZEB1. Moreover, in parent HuCCT-1 cells, co-transfection with a SPRR2a vector could significantly antagonize P300 induced promoter activation (**Figure 3F**). This observation supports an opposing function for P300/PCAF and SPRR2a in affecting *miR-200c/141* transcription in HuCCT-1 cells.

### P300/PCAF complexes with and acetylates ZEB1 on the miR-200c/141 promoter to activate transcription

**Figure 4:**
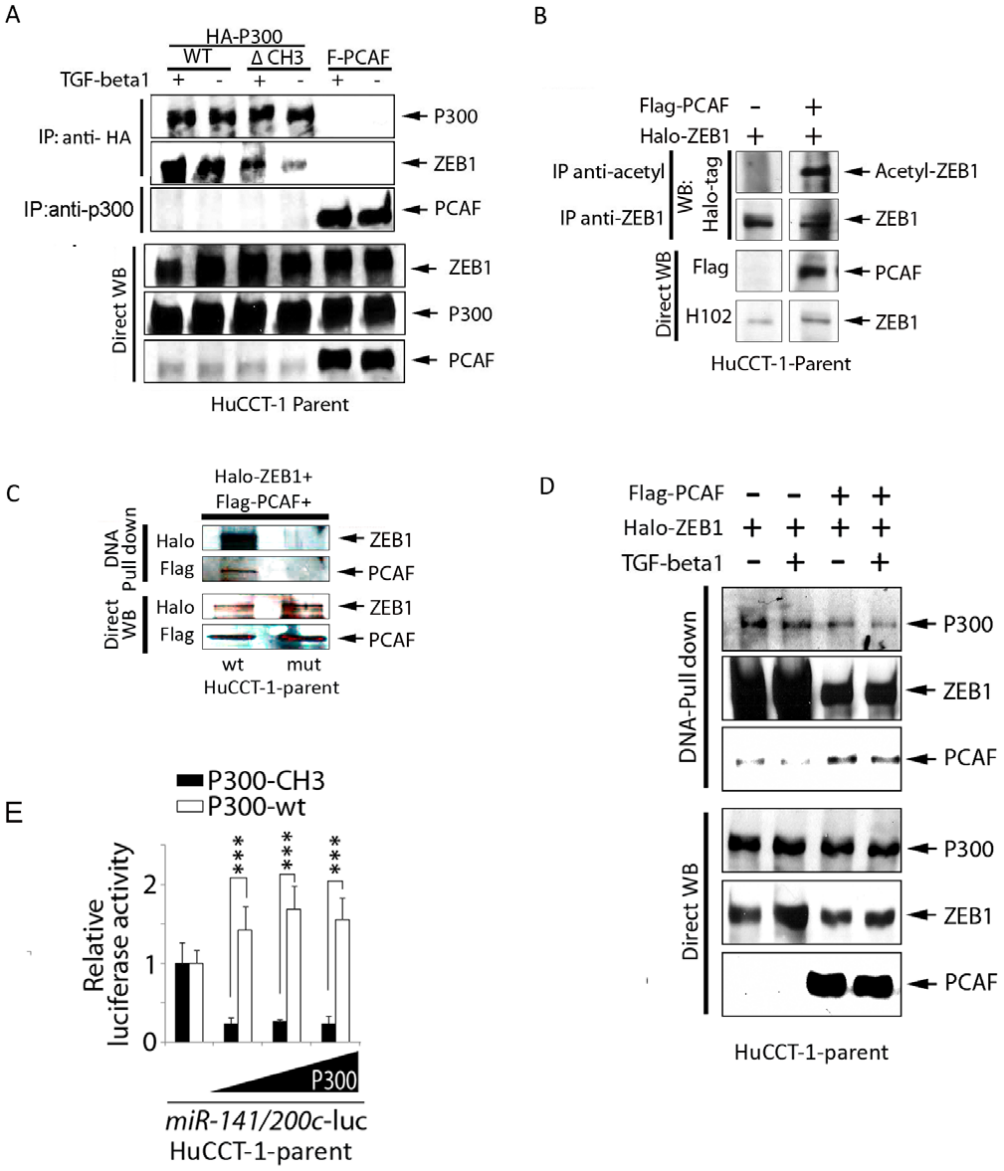
P300/PCAF complexes with ZEB1 on the *miR-200c/141* promoter and requires the CH3 domain of P300 for transcription. Immunoprecipitation of P300 following transfection with the indicated protein expression vectors verifies P300/ZEB1 and P300/PCAF interactions, which were unaffected by TGF-β1 treatments (5 ng/mL; 24 hrs) (**A**). Immunoprecipitation experiments show PCAF acetylates ZEB1 following transfection with ZEB1 ± PCAF expression vectors (24 hrs) (**B**). DNA pull-down assay using a wild type (wt) or mutational E-box/Z-box sequence for the *miR-200c/141* promoter after co-transfection of HuCCT-1 shows binding of ZEB1 and PCAF to the wt promoter sequence (**C**), and p300/PCAF/ZEB1 binding to the wt promoter, which is unaffected by TGF-β1 treatments (5 ng/mL; 24 hrs) (**D**). Luciferase assay for *miR-200c/141* promoter activity following transfection with wild type or CH3 deleted P300 expression vector in HuCCT-1 parent cells shows the CH3 domain is required for miR transcription. (n = 3 independent experiments; ***, *P*<0.001; Student's *t* -test) (**E**).

Co-immunoprecipitation of ZEB1 and P300 was seen in HuCCT-1 cells transfected with a HA- tagged P300 vector. In addition, less ZEB1 co-immunoprecipitates when a CH3 deleted P300 vector is used, indicating a role for the CH3 region during ZEB1/P300 interactions. Likewise, PCAF/P300 complexes were verified in HuCCT-1 cells transfected with a PCAF vector and immunoprecipitated using a P300-specific antibody. Finally, this binding of P300/PCAF/ZEB1 is not affected by TGF- β1, as was seen in a previous report [24] (**Figure 4A**).

HuCCT-1 cells, transfected with both PCAF and ZEB1 expressing vectors, show acetylation of lysine residues on ZEB1, whereas cells transfected with only ZEB1 have no lysine acetylation (**Figure 4B**). This observation is in accordance with a previous report [24]. To further investigate whether these interactions directly contribute to miRNA 200c/141 transcription, we constructed biotinylated double-stranded oligonucleotide probes that mimic the wild type or mutational sequences in the E-box and Z-box binding sites for ZEB1 (**Figure 3A**). Lysates from cells are incubated with the biotinylated DNA sequences and the resulting complexes precipitated with strept-avidin beads. Precipitating proteins are then visualized via western blot using appropriate antibodies. Lysates from HuCCT-1 parent cells, which were co-transfected with PCAF and ZEB1 expression vectors, show that ZEB1 and PCAF can only complex to promoter sequences with intact, wild type E-box/Z-box elements (**Figure 4C**). Moreover, P300 is also a component of the ZEB1/PCAF complex on the promoter (**Figure 4D**). A previous report showed ZEB1 switches from a transcriptional repressor to a co-activator in response to TGF-β1treatment [24]. In our cells, however, binding of P300/PCAF/ZEB1 is not affected by treatment with TGF beta1 (**Figure 4A and 4D**).

### The CH3 region of P300 is required for miR-200c/141 promoter activity

CBP and P300 were originally identified as factors binding to the cAMP response element-binding protein (CREB) [25] and the adenovirus E1A associated protein [26]. Numerous reports demonstrate that CBP/P300 have specific areas that allow for interactions with a wide array of transcription factors and co-factors [27]. The cysteine and histidine-rich region 3 (CH3) of P300 is one site for interaction with many transcription factors, including the adenovirus E1A oncoprotein, the co-activator PCAF and the SV40 large T antigen [26] [28], [29]. As shown in **Figure 4E**, transfection with a CH3-deleted P300 vector dramatically suppressed the activity of our luciferase miR-200c/141 promoter construct when compared to transfection with wild type P300. Without the CH3 region, there is less interaction between P300 and PCAF and reduced miR-200c/141 promoter activity. Also, immunoprecipitation of HuCCT-1-parent cells transfected with a CH3 deleted P300 vector (**Figure 4A**) yielded less ZEB1, indicating that P300 may need PCAF binding to fully interact with ZEB1.

### Changes in miR200c/141 and P300 can shift EMT marker expression

**Figure 5:**
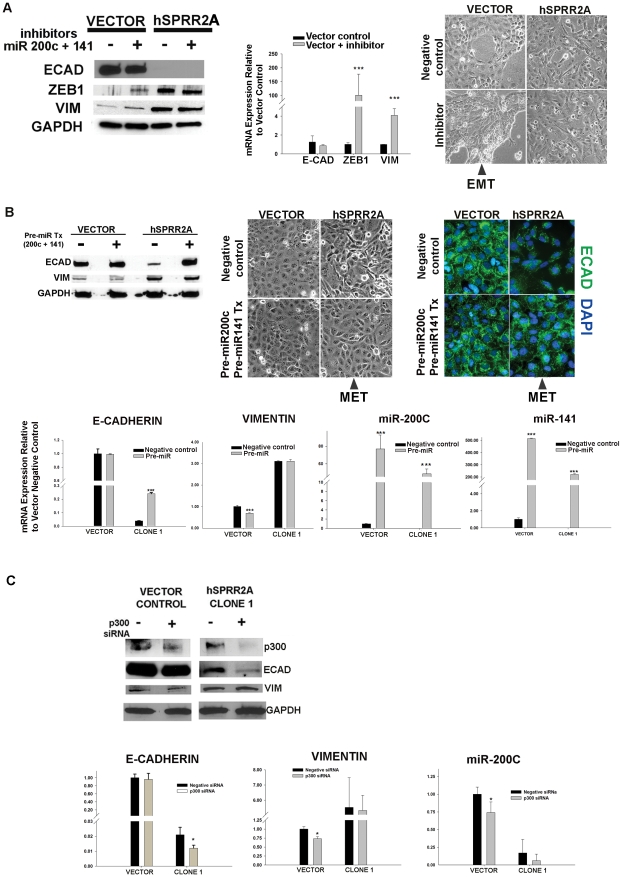
Changes in miR-200c/141 and P300 result in expected EMT/MET responses. Representative western blot and corresponding real time PCR analysis shows inhibition of miR200c/141 causes partial EMT shifts in vector cells as evidenced by increased Vimentin, increased ZEB1 and phenotypic changes. No change is seen in E-cadherin expression. (84 hr post transfection; cells grown on glass coverslips; n≥2 independent experiments) (**A**). Representative western blot (48 hr) showing partial MET in SPRR2A cells (increased E-cadherin; phenotypic changes on tissues culture plastic) following transfection with pre-miR-200c and pre-miR-141. Western blot results were verified by real time PCR analysis and immunofluorescence staining (n = 2 independent experiments) (**B**). Representative western blot (48 hr) and real time PCR showing changes in EMT markers following knock down of P300. Phenotypic changes were not observed, but EP300siRNA significantly reduced vector cell miR200c expression. (n≥2 independent experiments) (**C**) Real time PCR analysis: comparative 2−ΔΔCT method (U6 or GAPDH internal control); *, *P*<0.05; **, *P*<0.01; ***, *P*<0.001; Student's *t*-test).

First, to demonstrate the prominent role miR200c/141 has on the epithelial/mesenchymal state in HuCCT-1 cells, we measured how inhibition and overexpression of these miRNA changed EMT marker expression. Since miR200c/141 is transcribed en-block [11], we knocked down expression by transfection with dual inhibitors (Anti-miR™ 200c and 141). Successful inhibition was verified by monitoring target gene (ZEB1) expression. Inhibition of miR200c/141 yielded a partial EMT conversion in the vector cells, as evidenced by increased ZEB1 and vimentin accompanied by a phenotypic change (**Figure 5A**). This phenotypic change appears to be matrix dependent, as it was only observed when cells were grown on glass coverslips and not on tissue culture plastic. Despite these changes, vector cell E-cadherin expression was unaffected by the inhibitors. Real time PCR analysis correlates the changes seen in vector protein with mRNA levels (**Figure 5A**).

In a reverse experiment, dual transfection with pre-miR-200c and pre-miR-141 caused an MET shift in SPRR2a expressing cells. A significant phenotypic conversion was accompanied by an increase in E-cadherin expression (protein and mRNA), with localization along cell-cell borders. However, no changes in vimentin expression were observed (**Figure 5B**). Increases in mature miR-200c and miR-141 transcripts in both cell lines verified successful transfection with pre-miR species (**Figure 5B**).

EMT shifts in vector cells (Figure 5A) and MET shifts in SPRR2a cells (Figure 5B) verifie the central role of miR200c/141 in modulating cell phenotype. Alone, these agents caused significant changes in vimentin, E-cadherin, miR200c, and gross phenotype, depending on the direction of the transition, but not all 4 parameters changed simultaneously. This observation substantiates the viewpoint that EMT/MET is a complex process and cellular transition from one state to the other encompasses a continuum of phenotypes involving many effectors.

The role of P300 in EMT is controversial. Some show high P300 expression leads to EMT [30], while others show P300^−/−^ cells undergo EMT [7]; the differences might be contextual [1]. Since we showed that P300 forms a complex (P300/PCAF/ZEB1) on the miR200c promoter, resulting in lysine acetylation of ZEB1 and transcriptional activation of miR200c, we expected that knockdown of P300 would lead to a more mesenchymal phenotype (EMT shift) in our cells. As seen in **Figure 5C**, SPRR2a expression does not affect endogenous P300 protein levels. In our cells, P300 siRNA treatment yielded a significant decrease in miR200c expression for the vector cells (EMT shift). This is most likely due to a corresponding decrease in p300 mediated acetylation of ZEB1 on the miR promoter. Also, P300 siRNA did not significantly affect E-cadherin expression in the vector cells, but did decrease it in the SPRR2a clone (an EMT shift). P300 is a transcriptional co-activator that also binds to the promoter region of E-cadherin and enhances gene expression [31]. Reduced E-cadherin in the clone, but not the vector cells, suggests that SPRR2a over expression may affect the intrinsic acetyltransferase activity of P300 (an area for future study). Finally, vimentin expression was unchanged in the SPRR2A clone, but it unexpectedly decreased in the vector control. This suggests that P300 is not essential for maintenance of vimentin expression during SPRR2a EMT, but may affect gene expression in the epithelial state. Although P300 is involved with transcriptional control of miR200c, inhibition of this co-factor alone was insufficient to cause a phenotypic shift in our cells.

**Figure 6:**
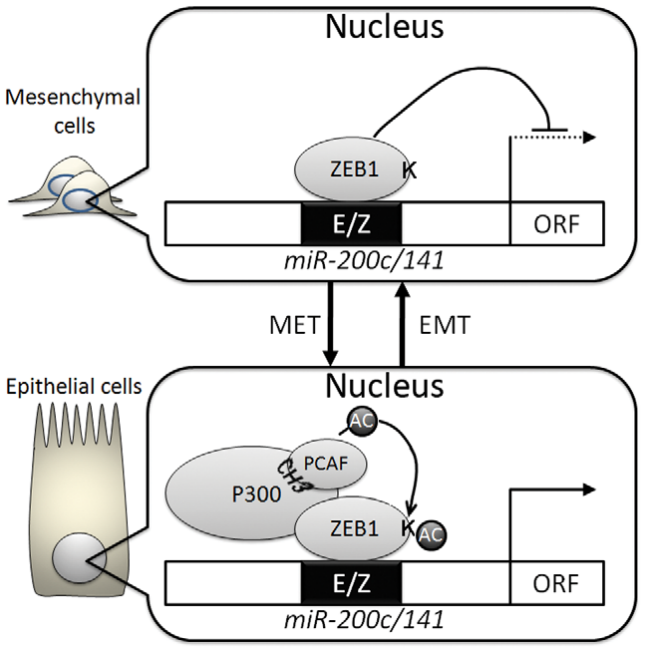
A Putative model illustrating the interactions of P300/PCAF/ZEB1 in modulating cell phenotype through control of *miR-200c/141* transcription. (AC = acetylation).

Transcriptional suppression of miR-200 family by ZEB1/ZEB2 is a central contributor to maintaining mesenchymal characteristics as well as inducing EMT [9]. Here, we show that P300/PCAF are cofactors for ZEB1 capable of reversing the ZEB1 suppressive effects on miR-200c/141 transcription and the important role these miRNA have on determining cell phenotype. This is a newly discovered mechanism for miR-200c/141 transcriptional regulation. Also, this data implicates P300/PCAF in controlling cell phenotypes through regulation of miRNA expression. In **Figure 6**, we proposed a model to illustrate how P300/PCAF controls epithelial/mesenchymal characteristics through modulating ZEB1 suppression of the miR-200c/141 promoter. To activate *miR-141/200c* transcription, P300 binds to PCAF through its CH3 domain, which in turn, facilitates miR-200c/141 transcription, whereas disruption of the P300-PCAF (Figure 4E) interaction strongly suppresses the promoter activity. One mechanism for P300/PCAF activation of *miR-200c/141* transcription is through lysine acetylation of ZEB1 (**Figure 4B**), which is concordant with the previous report showing that acetylation of ZEB1 interrupts CtBP binding, and prevents ZEB1 from acting as a transcriptional suppressor. It also explains how TSA treatment in SPRR2a cells can enhance miR200c/141 expression (**Figure 2A**). Although TGF-β1 has been shown to contribute to EMT [32], in HuCCT-1 cells, TGF-β1 did not alter P300/PCAF/ZEB1 binding on the miR200c promoter (**Fig. 4A and 4D**).

Our data verifies P300/PCAF/ZEB1 interactions on the miR-200c/141 promoter and the importance acetylation plays in transcriptional regulation of this miRNA. Forced acetylation (via TSA) and manipulation of P300, PCAF, ZEB1 and SPRR2a expression (via transfection) can shift miR200c promoter activity and EMT marker expression. How SPRR2a affects the interaction of P300/PCAF/ZEB1 followed by EMT or how cells regulate P300/PCAF itself is still an open question and likely involves other cofactors yet to be explored. Our findings show a novel and unifying mechanism for the effect of acetyltransferases on miRNA transcription and the potential for morphological consequences.
